# An analysis of COVID-19 economic measures and attitudes: evidence from social media mining

**DOI:** 10.1186/s40537-021-00431-z

**Published:** 2021-03-01

**Authors:** Dorota Domalewska

**Affiliations:** National Security Faculty, War Studies University, Al. Gen. Chruściela Montera 103, 00-910 Warsaw, Poland

**Keywords:** COVID-19, Economic attitudes, Social media, Relief package, Pandemic, Big data, Social media mining

## Abstract

This paper explores the public perception of economic measures implemented as a reaction to the COVID-19 pandemic in Poland in March–June 2020. A mixed-method approach was used to analyse big data coming from tweets and Facebook posts related to the mitigation measures to provide evidence for longitudinal trends, correlations, theme classification and perception. The online discussion oscillated around political and economic issues. The implementation of the anti-crisis measures triggered a barrage of criticism pointing out the shortcomings and ineffectiveness of the solutions. The revised relief legislation was accompanied by a wide-reaching informative campaign about the relief package, which decreased negative sentiment. The analysis also showed that with regard to online discussion about risk mitigation, social media users are more concerned about short-term economic and social effects rather than long-term effects of the pandemic. The findings have significant implications for the understanding of public sentiment related to the COVID-19 pandemic, economic attitudes and relief support implemented to fight the adverse effects of the pandemic.

## Introduction

The COVID-19 pandemic is an unprecedented crisis that is having a major impact on state and international security. The fast spread of the virus has caused many hospital admissions and a rapidly increasing death toll. A near global lockdown was implemented and although this has been partially lifted since May 2020, the pandemic is likely to have far-reaching consequences, the eventual scale of which is impossible to predict at the moment (November 2020). Stay-at-home orders are being issued globally and social distancing is being promoted to reduce the risk of contracting the disease. Undoubtedly, the consequences of the pandemic surpass the impact on public health and health care systems and will have a massive impact on state and international security. Multiple policy interventions all over the world were launched to tackle the crisis, including imposing national lockdowns and closure of retail businesses. Various countries adopted different strategies to deal with the outbreak of the crisis (March–April 2020). Some countries, e.g. Italy, implemented a total lockdown, several other countries, e.g. the UK, reduced the functioning of businesses and services, whereas a few countries, e.g. Sweden, employed a relaxed strategy based on merely promoting social distancing without shutting down or reducing business operations. The short-term effects of socio-economic restrictions on the epidemiological situation have been partially effective [1]. Unfortunately, the long term consequences of the mitigation measures will have unprecedented long-term effects, in particular on education [2], the level of crime and violence [3, 4], an overloaded healthcare system, costly social safety nets, and mass unemployment and business failure [5]. The global lockdown and social distancing measures have precipitated a social crisis [6] and an economic downturn [5, 7].

Since there is little published data yet on the public perception of economic measures implemented to fight the adverse effects of the pandemic, this paper investigates communication on two social networking sites, Facebook and Twitter, to explore public opinion of the relief package launched in the face of the COVID-19 pandemic. The study seeks to answer the following research questions: How do social media users evaluate COVID-19 economic measures? What factors affect the perception of economic measures? We focus on social media discussion about the relief package introduced by the authorities in Poland at the outset of the pandemic (March–June 2020). We seek to make two contributions to the literature on this topic. Firstly, the study is important because research on the polarity of economic news during the COVID-19 related economic slowdown provides important clues both to the authorities (on how to improve legislation) and to journalists (on how to frame the news to prevent crisis development). Secondly, given that the framing of the crisis has a significant impact on economic and political performance, our findings are important. Negative economic attitudes make the financial market more susceptible to recession and slow down economic recovery. The consequences of lingering negative sentiment may activate “a mutually reinforcing cycle in which consumer behavior becomes detached from economic reality” [8]. When economic outlooks turn gloomy, consumer spending is likely to decrease, the economy may contract, and media coverage becomes more negative, which further generates an atmosphere of negativity and reduces consumer spending even more.

## Economic measures in the face of the COVID-19 pandemic

Risk management is a systematic approach incorporated by every enterprise to manage uncertainty in order to satisfy stakeholders’ needs. The risk management process is crucial for developing company strategy and control. It is a dominant driver for value creation, competitiveness and profitability [9]. Effective risk management entails planning, activating, directing and controlling resources to achieve the company’s objectives in the face of unexpected circumstances. As a result of the differences in its role and function, businesses and governments have different requirements for risk management and mitigation. As Wang and Pei point out, “enterprises are often concerned about risks that describe concrete hazards in the working area. After evaluating the risk level of each hazard, those at higher levels are prioritised for greater control. Instead of treating individuals solely as management objects, for supervision convenience, governments care more about risks that measure the overall safety of a group of hazards in the jurisdiction. This regional risk makes it easy for safety supervisors to scientifically determine regulatory direction and reasonably allocate regulatory resources” [10]. Therefore, new approaches are required to deal with risk and the economic cost of risk management and disaster prevention.

The pandemic has generated unprecedentedly high economic costs. In the face of the pandemic and recession, the whole world has implemented strict regulatory measures. Central banks, authorities and international organisations have launched numerous interventions to stimulate the economy [11]. The International Monetary Fund assessed that the Australian government’s economic support package of AUD 320 bn was introduced to support households and businesses [12] whereas the loans, equity injections and guarantees are worth USD 4.5 trillion [11]. The relief package in the US amounts to USD 2 trillion. The European Union spent €500 bn to rescue European countries suffering from the pandemic [13]. Poland added measures worth €49.24 bn to a relief package. Central banks all over the world reduced interest rates and relaxed countercyclical capital buffers for numerous financial institutions.

According to the World Bank forecast, Poland is bound to experience a massive slowdown; however, the country has fiscal and monetary resources to mitigate the detrimental effects of decreasing global and domestic demand and protect the population at risk. Poland introduced a series of measures to mitigate the consequences of the economic crisis. On March 31, 2020, the Polish government launched new legislation [14], the so-called anti-crisis shield, which was approved in order to curb detrimental market effects. The bill introduced government subsidies for enterprises experiencing a reduced turnover, extended the deadlines for foreigners to submit applications for work and residence permits, released enterprises with fewer than 10 employees from social security premiums, and offered a one-off standstill allowance. Furthermore, the regulation offered flexible working arrangements, alterations to tax regulations, and the possibility of fixing maximum prices and margins for the sale of goods and services related to human health or safety and household maintenance goods. Finally, the prohibition on Sunday trade was suspended for trade-related activities. The anti-crisis programme had been amended twice at the time of writing the article. The anti-crisis shield 2.0 introduced on April 16, 2020 [15] offered companies new support from the Industrial Development Agency in order to improve their financial liquidity, increased the number of entrepreneurs entitled to exemption from social security premiums, increased the number of micro entrepreneurs eligible for loans, and extended the right to a standstill allowance. The bill that came into force on May 14, 2020 [16] amended the relief package, and also extended the additional allowance for carers of a disabled child or adult, expanded the right to be exempted from social security premiums, increased amounts free from deductions from remuneration, and extended the rights to a standstill allowance. The bill introduced significant changes for foreigners, such as allowing them to commence seasonal work without the need to obtain a permit and to work under amended conditions without needing to renew their permit.

A growing body of literature has been examining the impact of the pandemic on the global economy. Baker et al*.* [17] and Zhang et al*.* [5] analysed the effects of the crisis on the aggregate markets. Global economic growth declined sharply in response to the pandemic. The uncertainty caused by the pandemic and resulting economic losses has led to financial markets experiencing volatility and unpredictability. As individual countries are taking different national-level measures to deal with the crisis, the long-term adverse effect is related to the disintegration of the global community. McKibbin and Fernando [18] and Akhtaruzzaman et al*.* [11] investigate the effect of the health crisis on the global economy. International stock markets and foreign exchange markets are more risky for international investors since the crisis began. Coherent cross-national regulations need to be introduced to prevent market participants (especially banks) from reducing their risk-taking capacity and to support liquidity on international markets. Sharif et al*.* [19] investigated the correlation between the COVID-19 pandemic, the crude oil price volatility shock, the economic policy uncertainty, the geopolitical risk and the US stock market. The researchers argue that the long-term effects of the pandemic are related to high geopolitical risk levels and economic uncertainty, including a slump in oil prices. The effects of a significant plummet in oil prices will exert influence not only on oil exploration and production, but also the transport and hospitality industries. Governments need to take coherent measures for opening markets to avoid causing uncertainty.

## Social media and economic attitudes

Social networking sites are a lens through which public sentiment can be easily accessed. A large body of research proves that social media data can be mined for a variety of reasons. Public sentiment displayed on social media can be used to predict the stock price of companies [20], highlighting areas of focus for public health [21], and can affect political participation [22] and political opinions [23, 24]. Social media is also priceless in risk management and mitigation [25, 26].

With the exponential growth of user-generated content published on social networking sites, a variety of actors (businesses, governments, and researchers) have turned to data mining. Social media mining includes an array of analyses, from simple counting of the likes, retweets and users’ demographics to more sophisticated measuring of quantifiable information such as sentiment, popularity or reach. Data mining techniques encompass social network analysis, Bayesian networks, decision trees, natural language processing, and other algorithms.

Sentiment analysis examines the valence of social media data. The computation of the orientation of a post or tweet usually takes two main approaches: semantic orientation and machine learning. The semantic orientation approach is a rule-based linguistic model that extracts the sentiment from a text segment. This approach requires the creation of a dictionary of terms and their polarity value. The machine learning, on the other hand, is based on model creation by using annotated or weakly labeled data. Both methods produce relatively accurate polarity values. Dictionaries, such as SentiWordNet, are available in English; for other languages, the creation of individual sentiment lexicons is necessary. Furthermore, the context of the message, e.g. irony, is not taken into consideration. On the other hand, machine learning requires prior training; it also depends on the availability of labeled training datasets which are difficult to attain.

Social media mining has become a popular method of research because social networking sites are the key communication platforms that are used universally for public debate [27–30], which in turn facilitates day-to-day operations and communication of public institutions [31–33]. Few studies, however, employed data mining for economic-related posts, tweets, and news. Salas-Zarate et al. [34] employed an ontology-driven approach to semantically analyze relations between concepts in financial news. The researchers tested the accuracy of the experimental technique of opinion-mining; therefore, they were focused on the method of polarity classification rather than the sentiment of news items. Recent studies [8, 35] prove a strong correlation between public economic attitudes and economic performance showing that economic attitudes drive economic behavior, whereas other researchers [36–38] provide evidence of the impact of the positive or negative bias in news on financial markets. The framing of the news played a significant role in share prices and precipitating economic downturn. These studies, however, investigated the traditional mass media. An important study was carried out by Soroka et al. [39] who found that the traditional media frames economic news with a negativity bias, whereas social media exhibits a positivity bias which, in turn, has a direct effect on a shift in economic and political behavior. Cerchiello and Giudici [40] analyzed financial tweets to prove that the Twitter feed can be used to estimate systemic risk networks, whereas Nofer and Hinz [41] showed that Twitter sentiment can be used to predict German stock markets.

The abovementioned studies prove that the sentiment of social media users accurately predicts a range of behaviors. The pandemic resulted in increased public concern. COVID-19 risk perception has intensified due to several terrifying factors, such as the contagiousness of the infection, fast rising morbidity and mortality, and insufficient measures for infection prevention and control. In a study on risk perceptions of COVID-19, Dryhurst et al*.* [42] found that at the beginning of the pandemic (March–April 2020), the risk perception of the threat was high in countries across Europe, Asia, and America. A multitude of interplaying social and personal factors increased the public perception of risk, especially experience, values and distrust of institutions. Alomari et al. [43] investigated the reasons for public concern as expressed by social media users. The researchers identified the following reasons for increased concern: lockdown measures, financial challenges, worries about social and environmental sustainability, and daily livelihood issues. Li et al. [44] focused on examining COVID-19 related stress symptoms. The researchers found a correlation between increased risk perception and media coverage of the pandemic. The concern of social media users shifted from health-related fears (at the beginning of the pandemic in January through March 2020) to financial challenges. Thus far, there is limited research on the polarity of economic posts and tweets. This study aims to fill this gap by examining a sample of social media feeds published during the COVID-19 pandemic.

## Sampling and method

A large data set was investigated in order to determine public perception of economic measures implemented at the outset of the COVID-19 pandemic. The study aims to answer the following research questions: (1) How do social media users evaluate COVID-19 economic measures? (2) What factors affect the perception of economic measures? In order to answer the research questions, both quantitative analysis and content analysis were employed. Two variables were examined in order to determine the public perception of economic support offered by the government: the number of infected cases and online media coverage.

We collected data from Facebook and Twitter, as these platforms are the most popular social networking sites in Poland that allow users to express their opinions on a variety of topics. Furthermore, relevant tweets and posts can be easily categorised through the use of hashtags and key words. We used Twitter Streaming API to collect tweets and Unamo social media mining software to harvest Facebook posts and news alerts published by media companies. All data was collected from public domains; therefore, it was not necessary to obtain user permission to conduct the analysis. Both metadata and textual features of mentions were collected. Metadata refers to the characteristics of the mention, such as user id and publication date. Textual features are the content of the tweet or post. The corpus contains 666,495 tweets and Facebook posts and 9,746 online news items harvested through a combination of hashtags and keywords associated with the impact of the pandemic in Poland in the period between 4 March and 24 May 2020. 4 March 2020 was the day when the first confirmed case of COVID-19 was reported in Poland. The hashtags through which the corpus was collected were actively used in the public debate on social networking sites. They referred to the economic effect of the pandemic and instruments implemented to mitigate these effects (#anti-crisis shield; #anti-crisis measures; #support; #shield; #COVID-19). Selecting mentions on the basis of hashtags offers an opportunity to gather the most relevant content related to COVID-19.

Having collected the corpus, the next stage involved pre-processing and cleaning tweets and posts. This was an indispensable stage in creating a dataset because the presence of noise in a sample may result in inaccuracy. First, the data was filtered to remove redundant posts. The corpus had to be screened as it contained numerous items of marketing information or political content that was not directly related to the economic impact of the pandemic. Next, the data was cleaned using Python. Cleaning entailed removing the Twitter handle and Facebook username, lemmatization and tokenization as well as removing punctuation, stop words, special characters, and duplicate entries.

Next, the corpus was analysed quantitatively. Descriptive statistics were used to calculate frequencies, means and standard deviations (SD). The linear regression was carried out to identify a correlation between the number of posts and two variables: the number of confirmed cases of COVID-19 reported in Poland and the number of news published online by media companies.

A qualitative analysis was carried out on 33 325 tweets and posts (5% of the corpus). These entries were selected for the dataset if they contained at least one keyword from two prefixed lists of keywords (list 1: COVID-19, pandemic, coronavirus; list 2: economy, shield, anti-crisis, support, financial, entrepreneur, businesses). The post or tweet was therefore selected for qualitative analysis if it contained at least 2 keywords, which limited the size of the sample. The lists of keywords ensured the relevance of posts, i.e. all of them referred to the economic effects of the pandemic. The dataset was analysed with C# media analytic tool to identify dominant themes associated with the economic impact of the pandemic. The detection algorithm was able to find the key words in any part of the post or tweet.

The sentiment analysis, i.e. the method used to examine the valence of social media data, was performed using lexicon-based analysis (sentiment analysis was described in detail in the previous section). This method gauges the sentiment of the words and phrases from the dataset based on their semantic orientation in a dictionary. Since the dataset was in Polish, we could not rely on available resources. Instead, we applied a three stage-based methodology that included manually constructing dictionaries, classifying data, and applying the prediction algorithm [45]. In order to build positive, neutral and negative dictionaries, we defined the semantic orientation of 3280 lexical items in Polish, mostly adjectives and verbs. Many of the items were downloaded from publicly available onthologies and then translated into Polish. Next, the sentiment score was calculated for the dataset: tokens were aligned with lexicon items that contained a sentiment label. Each word in the lexicon was assigned a value of 1 if they pertained to a positive or negative valence, or 0 if they did not pertain to that valence. The total positive and negative valence for each tweet or post was gauged by calculating the cumulative score of positive and negative words from the sample. The analysis was carried out with a custom designed analytic tool in C#. This approach resembles [45, 46], which analyzed non-English datasets.

## Results

**Fig. 1:**
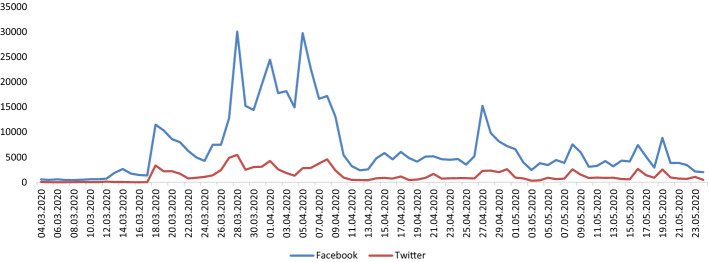
Intensity of channels by days

109,022 tweets and 557,473 Facebook posts related to the financial, business and economic effects of the pandemic were published during the study time frame, with an average 2,659 tweets and 6,798 posts per day. We reported a high degree of variation in the number of mentions depending on the publication date and channel (Fig. 1).

**Fig. 2:**
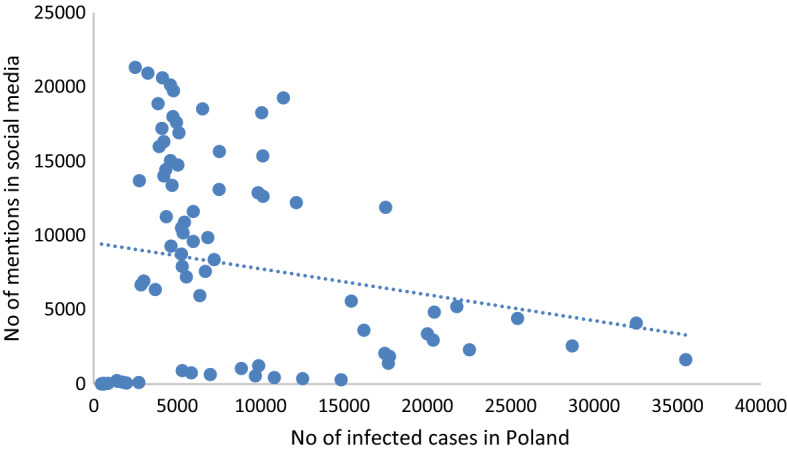
Correlation between the number of economy-related mentions on social media sites and the number of infected cases reported in Poland

**Fig. 3:**
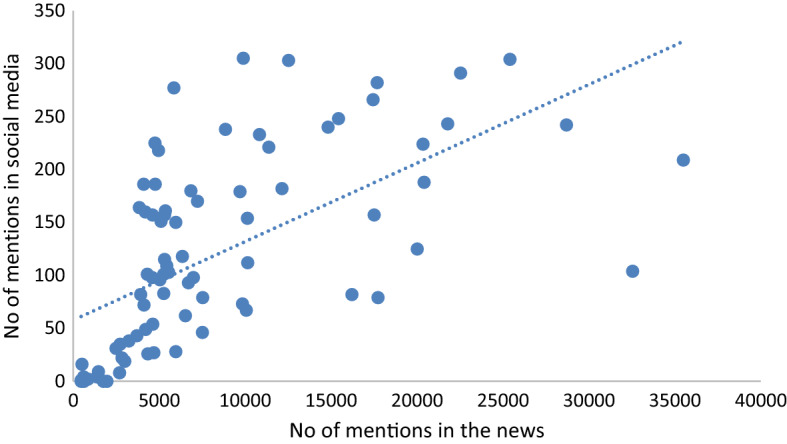
Correlation between the number of economy-related mentions on social media sites and the amount of economy-related online news

The presentation of longitudinal trends shows that the volume of Facebook posts was much higher than Twitter, which is related to the fact that Facebook commands the greatest share of mentions published on social networking sites in Poland [47]. The intensity of discussion reached several peaks during the study time frame that coincided with first the debate on the proposals of the relief package and then their implementation by the Polish government (the anti-crisis shield was first introduced on March 31, then updated on April 16, and May 14). The following Figs. 2 and 3 display the correlation between the intensity of discussion and two variables: the number of infected cases and coverage in online news.

The linear regression analysis showed a negative correlation between the number of economy-related mentions and the number of confirmed COVID-19 cases in Poland (R^2^ = 0.036) (Fig. 2), which shows that public perception of risk of economic instability was not associated with the steadily growing pandemic. Instead, a correlation was found between the number of economy-related mentions and the coverage of this topic on online news sites; however, the correlation was weak (R^2^ = 0.3782) (Fig. 3). This finding proves the considerable impact of the mainstream media coverage on discussion in social media. First, mainstream news is reposted or retweeted, which affects the volume of the mentions. Next, the media introduces the topics we discuss and shapes our reality.

**Fig. 4:**
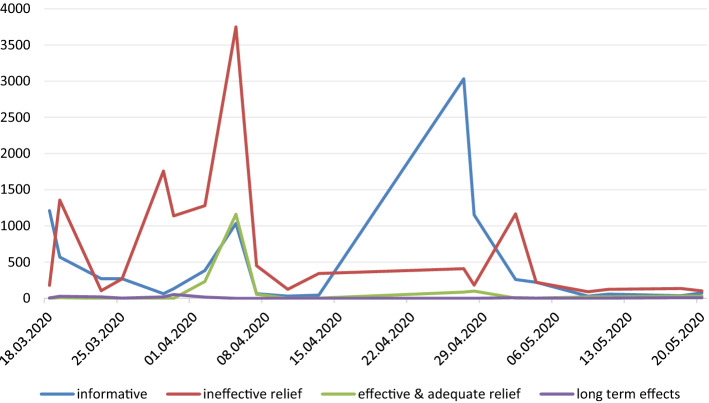
Intensity of discussion on the selected topics

Further analysis was carried out on 33,325 tweets and posts related to the economic impact of COVID-19. This dataset was analysed to identify the dominant themes. Four major themes were established: posts giving information on risk mitigation measures adopted in Poland (37.1% of the sample), posts pointing out the ineffectiveness of the implemented relief package (54.7%), posts indicating how effective and adequate the relief package was (7.3%), and posts identifying long term effects of the COVID-19 pandemic (0.7%). Figure 4 shows the timeline of the intensity of online debate on these topics.

The timeline clearly shows that the topic of the post corresponds to the relief package implemented by the government. At the outset of the pandemic, social media users posted comments demanding that the authorities introduce some risk mitigation measures that would counter the effects of the pandemic. When the first anti-crisis shield was implemented on March 31, citizens pointed out its shortcomings and ineffectiveness. The revised anti-crisis shield was launched on April 16 and May 14. At that time, informative posts prevailed as both the mainstream media and authorities launched a wide-reaching informative campaign about the relief package that was made available to companies, organisations, and individuals. The number of posts pointing out the effectiveness of the relief package rose immediately after the first anti-crisis shield was launched. These posts were also more frequently posted after the revised anti-crisis shield was implemented. The long term effects of the pandemic have not attracted much online attention. Table 1 shows the main discussion threads within each identified theme identified by qualitative analysis.

**Table 1:** Theme classification model

Topic and subtopics	Example post or tweet (translated into English)
Informative	There is a plan! The state will cover part of the salaries of employees, and pay the self-employed and part-timers who have been deprived of earnings due to the pandemic The Social Security Office / Taxes / Finances / Anti-crisis shield Check out what help your company can get
Ineffective relief	Entrepreneurs have no illusions since yesterday. They are disappointed with the anti-crisis package solutions announced by the government Leaky anti-crisis shield—companies point out shortcomings
Catastrophic effects of the pandemic	The shield doesn’t work. The labour market is collapsing. Apart from the self-employed, we have lost 153,000 full-time jobs. Salaries down, unemployment sharply up
Problems with administering the relief package	Entrepreneurs have been waiting for the funds due for the third month
Effective and adequate relief	The anti-crisis shield is adequate at the moment. It should shield the economy from the effects of the crisis
Personal story	A real anti-crisis shield. A landlord has reduced rent to 1 PLN. The florist—tenant praises him
Long term effects	Hard times are approaching. And I don't mean the pandemic time alone, but what happens afterwards. Total economic crisis, people out of work, and many companies going bankrupt. The country will be destroyed like after the war (…) What will happen to pensions after the pandemic? Pensions are another coronavirus victim. The provisions of the anti-crisis shield disturb the balance of public finances, taking about PLN 39 billion from the social security system. Forecasts for the coming months and years are not optimistic

The theme classification model presents topics that prevailed throughout the time frame of the study relating to the negative effects of the pandemic on the economy with the focus on short-term effects and demanding the authorities take some action. When the news of the relief package came out, social media users started to point out the weakness of legal solutions and problems with administering the relief package related to the delay in processing the application and payment of benefits. These posts prevailed for a fortnight during the period when the first anti-crisis shield was launched. They drastically declined just before the third anti-crisis shield was launched. A large volume of informative posts and tweets was posted for a fortnight during the period when the second anti-crisis shield was launched. These posts not only offered a general explanation of the bill but also gave detailed advice regarding specific problems entrepreneurs and employees might have had. Posts referring to effective and adequate relief fell mainly into two groups: posts with hyperlinks to mainstream media highlighting the effectiveness of the relief launched in the bill or personal stories posted by social media users who had been the beneficiaries of the relief package. Finally, a few posts referred to the long term economic effects of the pandemic. Posts in this group mainly concerned high inflation and budget deficit, negative impact on investment and pension schemes being under strain. Table 2 shows the results of the sentiment analysis.

**Table 2:** Sentiment analysis

	Total	%	Mean	SD
Positive	263,181	39.48	− 0.06	0.324
Neutral	374,469	56.18	− 0.07	0.312
Negative	28,845	4.32	− 0.12	0.285

The obtained results suggest that the neutral sentiment prevailed. Based on the results shown in Figs. 1, 2, 3, 4, it can be seen that the negative sentiment of messages prevailed during the first month of the study (March 4–April 7) when social media users published posts criticising the measures adopted in order to curb the spread of the virus. Overall, every fourth tweet or post had a positive sentiment, whereas negative entries made up 5.84% of the dataset.

Further analysis was carried out to identify the most common terms in the corpus. Two strands of terms were identified: economy-related terms and words related to politics and government. Economy-related terms included “shield”, “work”, “economy”, “anti-crisis”, “loan”, “tax”, “bank”, “rights”, which in the context of the COVID-19 pandemic can be categorised as risk-related terms. As these words prevail in the corpus, it can be concluded that the public are apprehensive of the economic and social risk of the pandemic and discuss risk mitigation schemes. The respect for human and civil rights of citizens is important in the face of the pandemic. The widespread occurrence of words related to politics and government (such as “Andrzej”, “Duda” [the name and surname of the incumbent president], “politics”, “government”) is connected, on the one hand, with the government being responsible for passing several bills but also with casting blame on the government and politicians for the disastrous effect of the pandemic. On the other hand, the pandemic broke out on the eve of presidential elections (that were held on June 28, 2020), which affected the social media discussion.

## Discussion

The findings of this study provide new insights into possible factors shaping economic attitudes. Contrary to other studies [48] which proved that the number of reported deaths of COVID-19 had a great impact on the public perception of the situation, this study proves that as long as economy-related issues are concerned, the growing number of reported COVID-19 infected cases does not correlate with the public perception. The content analysis of social media discussion showed that neither health issues nor public safety issues emerged in the analysis. Financial risk mitigation online discussion oscillated around political and economic issues. Informative posts prevailed in the dataset. These posts explained the bill providing the relief package (the so called anti-crisis shield) and gave advice about specific problems the public would face with administering or claiming the relief. Posts pointing to the negative impact of the pandemic on the economy were another major theme found in the sample. They either pointed out the short-term negative consequences, urged the authorities to actively fight the situation, or highlighted the ineffectiveness of the relief package. Some posts highlighted how effective and adequate the relief was or pointed out long term economic effects of the pandemic.

The study shows a weak correlation between the number of economy-related mentions and the coverage of this topic on online news sites. This finding shows the impact of the mainstream media coverage on discussion on social media platforms. First, mainstream news is reposted or retweeted, which affects the volume of the mentions. Next, the media decides the topics we discuss and this influences public opinion (the agenda-setting theory), which is in line with other studies [36–38]. The framing of the news affects not only public attitudes but also economic and political behavior. This study is also consistent with Soroka et al. [39] as social media posts and tweets exhibit significantly more positive bias than the news. We also found that the tone of the news broadcast on social networking sites is significantly more neutral than the tone of posts and tweets.

The analysis of longitudinal trends and the theme classification model prove the association with economic trends related to the implementation of the relief package by the government. At the outset of the pandemic, social media users posted comments demanding the authorities introduce some risk mitigation measures that would counter the effects of the pandemic. The implementation of the anti-crisis shield on March 31, 2020 triggered a barrage of criticism highlighting the shortcomings and ineffectiveness of the proposed solutions. The revised relief legislation was launched on April 16 and May 14 accompanied by a wide-reaching informative campaign about the relief package. This led to a reduction in negative sentiment. The analysis also showed that as far as economy-related discussion on risk mitigation is concerned, social media users are concerned more about short-term than long-term effects of the pandemic. Long-term concerns were mainly related to high inflation and budget deficit, negative impact on investment and putting pension schemes under strain.

## Conclusion

This study set out to investigate the public perception of economic measures implemented to fight the adverse effects of the COVID-19 pandemic. Social networking sites were mined to examine change in longitudinal trends and specify events that affect those trends. This study revealed that the online debate on COVID-19 economic measures oscillated around political and economic issues. The implementation of the anti-crisis measures triggered a barrage of criticism pointing at the shortcomings and ineffectiveness of the solutions. The revised relief legislation was accompanied by a wide-reaching informative campaign about the relief package, which decreased negative sentiment. The research has also shown that social media users expect the implementation of measures to support the security of the state and to mitigate the negative economic and social effects of the pandemic but maintaining respect for the rights of the employee.

The findings have significant implications for the understanding of public sentiment related to the pandemic, relief support and risk communication. Since there is little published data yet on the public perception of measures implemented to fight the adverse effects of the COVID-19 pandemic, this paper offers a significant contribution to the field of study. The research proves that social media helps to understand the public attitude [24, 40, 49]. Social networking sites are an important communication tool that can be used to disseminate information, affect the trajectory of negative sentiment, and promote credible and accurate news. Furthermore, since economic attitudes are correlated with political outcomes and negative economic attitudes make the financial market more susceptible to recession [8], the study has significant political implications. The polarity of the debate on the economic impact of the pandemic gives an insight for both the authorities, on how to improve legislation and develop best practices, and for journalists on how to frame the news to prevent economic slowdown. Increasing the number of positive posts and tweets may improve the economy [41] and the results of the study showed that launching an informative campaign about the relief package helped to reduce the number of negative messages. The research proved the effectiveness of social media data in detecting public sentiment and identifying events significant to users.

The major limitation of the study is that it has not provided clear evidence on the predictive value of tweets and posts. Furthermore, the distribution of sentiment analysis over time would provide accurate information on public opinion during different stages of the pandemic. More research is needed to further investigate the deeper meaning and correlations of the study results. A greater focus on regression analysis could produce interesting findings that account more for the explanation on how to utilise social media to promote global economic, health, and social objectives in the context of the pandemic.

## Data Availability

The datasets used and analysed during the current study are available from the corresponding author on request.
